# Linking brain activity during sequential gambling to impulse control in Parkinson's disease

**DOI:** 10.1016/j.nicl.2020.102330

**Published:** 2020-06-25

**Authors:** Brian N. Haagensen, Damian M. Herz, David Meder, Kristoffer H. Madsen, Annemette Løkkegaard, Hartwig R. Siebner

**Affiliations:** a Danish Research Centre for Magnetic Resonance, Centre for Functional and Diagnostic Imaging and Research, Copenhagen University Hospital Amager and Hvidovre, Hvidovre, Denmark; b Department of Neurology, Copenhagen University Hospital Bispebjerg and Frederiksberg, Copenhagen, Denmark; c Department of Applied Mathematics and Computer Science, Technical University of Denmark, Kgs. Lyngby, Denmark; d Institute for Clinical Medicine, Faculty of Health and Medical Sciences, University of Copenhagen, Copenhagen, Denmark

**Keywords:** Dopamine replacement therapy, Functional MRI, Gambling, Impulse control disorder, Parkinson’s disease, Reward

## Abstract

Dopaminergic treatment may impair the ability to suppress impulsive behaviours in patients with Parkinson's disease, triggering impulse control disorders. It is unclear how dopaminergic medication affects the neural networks that contribute to withholding inappropriate actions. To address this question, we mapped task-related brain activity with whole-brain functional magnetic resonance imaging at 3 Tesla in 26 patients with Parkinson’s disease. Patients performed a sequential gambling task while being ON and OFF their regular dopaminergic treatment. During a gambling round, patients repeatedly decided between the option to continue with gambling and accumulate more monetary reward under increasing risk or the option to bank the current balance and start a new round. 13 patients had an impulse control disorder (ICD + group). These patients did not differ in risk-taking attitude during sequential gambling from 13 patients without impulse control disorder (ICD - group), but they displayed differences in gambling-related activity in cortico-subcortical brain areas supporting inhibitory control. First, the ICD + group showed reduced “continue-to-gamble” activity in right inferior frontal gyrus and subthalamic nucleus. Second, the individual risk-attitude scaled positively with “continue-to-gamble” activity in right subthalamic nucleus and striatum in the ICD - group only. Third, ICD + patients differed in their functional neural responses to dopaminergic treatment from ICD - patients: dopaminergic therapy reduced functional connectivity between inferior frontal gyrus and subthalamic nucleus during “continue-to-gamble” decisions and attenuated striatal responses towards accumulating reward and risk. Together, the medication-independent (trait) and medication-related (state) differences in neural activity may set a permissive stage for the emergence of impulse control disorders during dopamine replacement therapy in Parkinson’s disease.

## Highlights

• Parkinson's patients with and without impulse control disorders gamble similarly.
• Patients with ICD have reduced activity in inhibitory control regions.
• STN and striatum activity scales with risk in patients without ICD but not with ICD.
• Dopamine therapy differently affects the groups' neural activity and connectivity.

## 1 Introduction

In Parkinson's disease (PD), dopamine replacement therapy (DRT) with dopamine D2/D3 receptor agonists and levodopa triggers various impulse control disorders (ICDs) in around 14–18% of patients (Weintraub et al., 2010, Joutsa et al., 2012, Poletti et al., 2013). The spectrum of ICDs comprises e.g., gambling, compulsive shopping, binge eating, hypersexuality, punding and hobbyism (Weintraub et al., 2015). ICDs are “behavioural addictions” with a failure to disengage from specific reward-seeking behaviours despite accumulating negative emotional and socio-economic consequences (Weintraub et al., 2015).

The emergence of ICD during dopamine replacement therapy on patients with PD has been attributed to two neurocognitive mechanisms (Mosley et al., 2019, Paz-Alonso et al., 2020, Cilia and van Eimeren, 2011). The first mechanism consists of functional alterations in the reward system, especially the ventral striatum (VS), which may alter reward valuation. DRT is adjusted to alleviate motor symptoms caused by a dopaminergic denervation of the posterior “motor” part of striatum. However, this treatment is hypothesized to inadvertently result in dopaminergic “overdosing” in anterior-ventral striatum involved in cognitive and limbic functions (Cools, 2006). Several imaging studies point to functional changes in VS consistent with a “hyperdopaminergic” state in patients with ICD: Tracer-based brain imaging showed a reduced density of dopamine transporters in VS (Cilia et al., 2010, Voon et al., 2014), and reduced striatal raclopride binding potential in the context of gambling and reward processing (Steeves et al., 2009, O’Sullivan et al., 2011, Wu et al., 2015). The latter points to increased endogenous dopamine release. Functional MRI (fMRI) of task-related brain activity revealed stronger neural responses to rewards, which might reinforce reward-seeking behavior (Voon et al., 2010, Politis et al., 2013).

Functional alterations in the inhibitory network constitute a second neurocognitive mechanism, which may contribute to abnormal impulsivity (Mosley et al., 2019, Paz-Alonso et al., 2020). Impulsivity is broadly seen as a failure of inhibition and is commonly divided into a “motor” subtype related to the failure to inhibit inappropriate actions and a “cognitive” subtype related to e.g. deferred gratification (Bari and Robbins, 2013). Patients with ICD are unimpaired in tasks testing motor impulsivity such as the stop-signal or Simon task (Wylie et al., 2012, Claassen et al., 2015), but impaired in tasks testing impulsivity in more cognitively demanding contexts such as temporal discounting (Housden et al., 2010, Voon et al., 2011), information sampling (Djamshidian et al., 2012), perceptual decision-making (Djamshidian et al., 2014), and novelty-seeking (Djamshidian et al., 2011). The functional brain alterations that underpin faulty cognitive inhibition in ICD have been associated with changes in a fronto-subthalamic network (Mosley et al., 2019, Paz-Alonso et al., 2020). This network consists of the pre-supplementary motor area (pre-SMA) and inferior frontal gyrus (IFG), which directly project to the subthalamic nucleus (STN) via so-called hyper-direct pathways, mediating the inhibitory control of action and cognition (Djamshidian et al., 2012, Djamshidian et al., 2014, Djamshidian et al., 2011, Aron et al., 2007, Jahfari et al., 2009, Neubert et al., 20107, Rae et al., 2015).

**Fig. 1:**
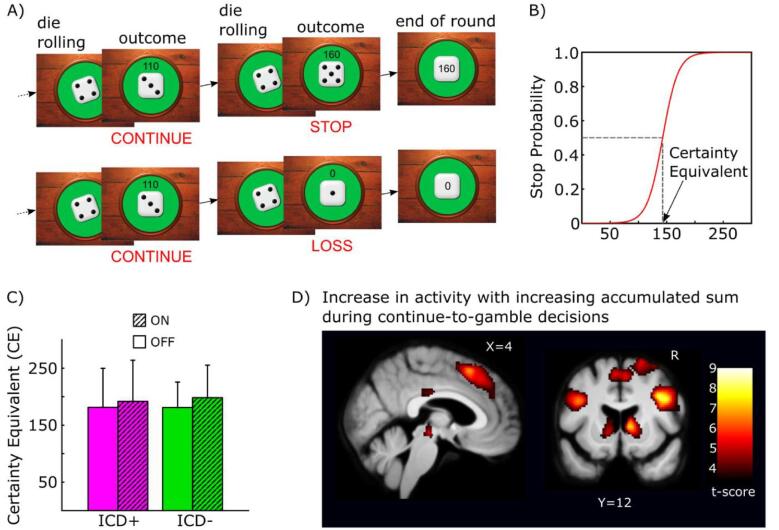
Experimental design, risk taking behaviour and brain activation during sequential gambling. A) Sequential gambling task. The task involved consecutive trials during which a die was thrown. The game consisted of “Stop”, “Continue”, and “Loss” events. B) Modelling of choice behaviour. Schematic drawing of the change in stop probability as a function of the accumulated sum earned during continued gambling. The certainty equivalent (CE) corresponds to the accumulated sum where the probability to stop is 0.5. C) Behavioral group data. Mean CE values in DKK for each PD group in the ON- and OFF-medication session (1 DKK ≈ 0.17 USD). The error bars equal one standard deviation. Please note that neither main effects of group and medication state nor the interaction between the two factors were significant. D) Colour-coded statistical t-score map of brain regions where “Continue” activity increased linearly with the accumulated sum acquired during sequential gambling. The map contains pooled data of both groups (ICD + and ICD -) and both medication conditions (ON− and OFF-medication). For further details see supplementary table 1.

This fMRI study was designed to test these two neurocognitive mechanisms. To this end, we mapped task-related brain activity with the blood oxygen level dependent (BOLD) signal, while PD patients with or without ICD played a sequential gambling task. In this task, patients repeatedly had to decide whether to continue accumulating monetary reward under escalating potential losses (Fig. 1). These decisions to continue instead of stopping the gamble thus involve increasing response conflict. We have previously shown in healthy individuals that this gambling task activates the inhibitory fronto-subthalamic network (Meder et al., 2016).

While ICD is generally associated with “hyperactivity” in the VS in the context of reward, the current study involves decisions with increasing potential reward, but concurrently increasing risk of loss. Two previous fMRI studies found decreasing activity in the VS of PD patients with ICD during risky decisions (Rao et al., 2010, Voon et al., 2011). Thus, we hypothesized that ICD would be associated with an attenuation of the valuation signal in VS during increasingly risky decisions. We additionally expected that ICD would be associated with diminished activation and reduced functional connectivity in the inhibitory fronto-subthalamic network, especially when patients are on medication. We also expected these neural differences to be coupled with increased risk-taking behaviourally.

## 2 Methods

### 2.1 Participants

We recruited age- and gender-matched PD patients with different sub-types of ICD and PD patients without ICD. We originally enrolled 42 patients with akinetic-rigid PD without cognitive impairment or contraindications regarding MRI, but we had to exclude 16 patients due to anatomical abnormalities, insufficient task performance and drop-out between sessions.

Data obtained from 26 patients entered the final group analyses (13 patients with clinically diagnosed ICD related to DRT (ICD + group)). Most patients had more than one type of ICD (Table 1). The remaining 13 patients served as disease controls (ICD - group). The study was performed in accordance with the declaration of Helsinki and informed consent was obtained from participants prior to engaging in the study. Experimental procedures were approved by the ethics committee of the Capital Region of Denmark (project ID H-3–2011-110).

**Table 1:** Demographic and clinical details of the PD patients. Between-group differences in gender and handedness were tested using chi-square test. The remaining between-group comparisons are based on two-sample t-tests using the total scores for each scale. MoCA: Montreal Cognitive Assessment; BIS: Barratts Impulsivity Scale; BDI: Becks Depression Inventory; QUIP: Questionnaire for Impulsive-Compulsive disorders in Parkinson's disease; LEDD: Levodopa Equivalent Daily Dose; UPDRS: Unified Parkinson's Disease Rating Scale.

Variable	PD patients with ICD (*n* = 13)	non-ICD (*n* = 13)	*p*(uncorr.)*
Gender	5 females	6 females	p = 0.43
Handedness (mean laterality index)	0.58	0.45	p = 0.64
Age (yrs)	59.4 (10.9)	61.4 (9.7)	p = 0.63
Education (yrs)	14.3 (3.5)	14.6 (2.7)	p = 0.80
MoCA	28.7 (0.9)	28.7 (1.3)	p = 1
BIS-11	59.5 (9.7)	55.8 (9.1)	p = 0.32
DOSPERT Risk	−0.4 (0.3)	−0.5 (0.3)	p = 0.33
BDI	7.4 (6.5)	7.0 (5.0)	p = 0.87
QUIP	5.1 (3.6)	0.5 (0.8)	p < 0.001*
Disease duration (years)	6.5 (3.6)	4.5 (2.0)	p = 0.08
LEDD (total)	694.5 (276.4)	569.6 (255.0)	p = 0.24
LEDD (agonist only)	221.6 (102.5)	203.5 (140.1)	p = 0.71
UPDRS-III-OFF	32.7 (6.1)	33.0 (8.6)	p = 0.92
UPDRS-III-ON	22.6 (6.0)	22.8 (6.9)	p = 0.65
UPDRS-III	11.1 (3.1)	10.2 (4.1)	p = 0.55
ICD diagnoses	Number of patients		
Excessive gambling	4		
Binge-eating disorder	9		
Compulsive Buying	4		
Compulsive sexual behaviour	7		
Hobbyism or punding	3		

### 2.2 Experimental procedures

Patients participated in three experimental sessions, separated by several days. The first session comprised an interview on the medical history, including impulsive-compulsive symptoms, questionnaires and neurological examination. Motor symptoms were assessed using the Unified Parkinson Disease Rating Scale (UPDRS-III). See Table 1 for the used questionnaires. The Danish translation of the “QUIP-current” (Weintraub et al., 2009) was back-translated to an English-language version and approved by one of the authors (D. Weintraub).

Whole-brain fMRI was performed during the second and third session. After a short training period, participants performed a 25-min gambling task during fMRI followed by structural MRI. Patients were scanned ON and OFF their normal DRT in a counterbalanced order at least two weeks apart. Before the OFF-session, patients paused DRT for a period corresponding to six drug half-lives. Dopamine agonists were gradually tapered off 3–4 days before MRI scanning. The daily levodopa dose was simultaneously increased until 12 h before the scans to maintain a similar level of levodopa-equivalent daily dose (Herz et al., 2014). Levodopa was then withdrawn 12 h before MRI scanning. Patients were told that they could contact clinicians (AL and BNH) via phone in case of disabling withdrawal symptoms.

### 2.3 Gambling task

The sequential gambling task (Meder et al., 2016) was presented on a screen visible through a coil-mounted mirror. Stimuli and recording of the subjects’ choices was controlled by PsychoPy software (v. 1.74.01, www.psychopy.org Peirce, 2009). The task comprised several gambling rounds. Patients repeatedly rolled a six-sided die with one to six pips on each surface (Fig. 1A). The number of each throw multiplied by 10 Danish Kroner (DKK, 10 DKK ~ 1.7USD) was added over trials. If the die showed “1”, patients lost the accumulated reward during that round. Patients were free to continue gambling or to stop and bank the accumulated reward. Since the accumulated reward and the potential loss gradually increased with each rewarding outcome, patients had to continuously trade-off between the prospect of foraging more reward and the risk of losing all accumulated reward by throwing a “1”.

Each trial started with a rolling phase where one random side of a 6-sided die was shown after each other for 150 ms. Rolling periods were jittered between 1.5 s and 3.5 s. At the end of the rolling period, the randomly drawn outcome was shown for 2 s, together with the accumulated gains. If the outcome was rewarding subjects had to press a PC-mouse button with their index or middle finger within 2 s to either continue the gamble or to stop the round and bank the total. The assignment of index and middle finger to continue and stop decisions was counterbalanced across patients. If subjects pressed “continue”, a new rolling phase started after 2 s. In case of choosing “stop” the banked amount was shown for 2.5 s and a new gambling round was started. In case of throwing a “1” (loss trial), a “0” was shown for 2.5 s. Thereafter, a new round began. The outcomes were randomly generated for each participant. Patients received the average outcome of all rounds as pay out in DKK at the end of each MRI session, including lost rounds with zero-outcome. This minimized opportunity costs associated with continuing to gamble and thus, incentivized participants to avoid the strategy of stopping each round after the first outcome. Given the relatively slow pacing of responses, the task is likely charging the more cognitive aspects of impulsivity (e.g. deferred gratification) rather than motor impulsivity (e.g. impulsive repetitions of the same response) (Bari and Robbins, 2013).

### 2.4 Magnetic resonance imaging

Whole-brain MRI was performed using a 3 T Verio scanner equipped with a 32-channel head-coil (Siemens, Erlangen, Germany). Whole-brain MRI was performed using a 3 T Verio scanner equipped with a 32-channel head-coil (Siemens, Erlangen, Germany). Image pre-processing was performed with statistical parametric mapping (SPM) software (version: SPM8 revision 4667; Wellcome Department for Neuroimaging, University College London). Structural T1-weighted images were acquired using a Magnetization Prepared Rapid Acquisition Gradient Echo (MPRAGE) sequence (TR 1900  ms, TE 2.32 ms, flip angle 9°). The field of view (FOV) covered the whole brain (FOV 230 × 230 × 230 mm) with a slice thickness of 0.9 mm. The T1 images were segmented into grey and white matter and cerebrospinal fluid compartments.

### 2.5 Analysis of choice behaviour

Logistic regression was used to model individual binary choice behaviour using the accumulated sum as predictor. At trial n*,* the probability of choosing the stop response was modeled as: $p\left(stop|x_{n}\right)=\frac{1}{1+exp\left(-w_{1}x_{n}-w_{0}\right)}$, where $x_{n}$ is the accumulated sum in trial n and $w_{0}$ and $w_{1}$ are free parameters. The “Certainty Equivalent” (CE) was defined as the amount at which the probability of choosing “stop” was 0.5. The individual CE was used to compare risk-taking attitudes between the patient groups (Fig. 1B). We also recorded reaction times on “continue-to-gamble” trials and related them to accumulated sum with linear regression using regression slopes as additional behavioural measure.

### 2.6 Analysis of functional MRI data

Functional brain imaging during the gambling task involved a T2*-weighted echo-planar imaging (EPI) sequence (TR 1.65 s, TE 26 ms, flip angle 74°) covering the whole brain. 32 slices per brain volume were acquired in ascending order with an in-plane resolution of 3 × 3 mm and a slice thickness of 3.2 mm (FOV 192 × 192 × 134.4, acquisition matrix 64 × 64). 910 brain volumes were collected in a single fMRI session. Pre-processing involved slice-time correction to TR/2, realignment to the mean EPI image of the first session, and co-registration to the individual T1-weighted structural MRI. Diffeomorphic Anatomical Registration using Exponentiated Lie algebra (DARTEL) (Ashburner, 2007) was used for normalisation of individual segmented T1-images. A group-specific template for overlay for functional images was constructed by averaging the individual normalized T1-images. The functional EPI scans were normalised to stereotactic space using the Montreal Neurological Institute template to an isotropic voxel size of 2 mm, using the individual grey matter-to-template flow-fields obtained from the DARTEL procedure. After normalisation, images were spatially smoothed using an isotropic Gaussian kernel with 8 mm full-width at half-maximum and temporally high-pass filtered at 1/128 Hz. Pulse and respiration were recorded during fMRI with an infrared pulse oximeter and a pneumatic thoracic belt.

Only one participant in the ICD + group once showed a slice-to-slice displacement of more than 3.0 mm (3.70 mm), and only very few patients showed slice-to-slice displacement of ≥2.0 mm (never more than 5 times per fMRI run). We compared head movements between the two groups using the method described in Power et al. (2012). Mean frame-to-frame displacement was below 0.4 mm in all but three fMRI sessions, confirming that patients had no difficulties to lay still during fMRI. Repeated measures ANOVA with medication status as within-subject effect and ICD-status as between-subjects effect revealed a significant effect of ICD-status (F = 7.84, p = 0.01). This was due to a subtle but significant increase in mean frame-to-frame displacement in the ICD + group relative to the ICD - group (ICD + group: 0.278 ± 0.118 (SD), ICD - group: 0.177 ± 0.056 (SD), post-hoc two-sample *t*-test: t_24_ = 2.800, p = 0.01). Medication status and the interaction between medication and ICD-status were not significant (F = 1.87, p = 0.18; F = 2.81, p = 0.11, respectively). Since the between-group difference in head movements was on average about 0.1 mm, we deem it unlikely that they affected the between-group comparisons.

Note that the lack of distortion correction might have reduced our sensitivity to BOLD signal changes, especially in basal forebrain regions (Hutton et al., 2002).

A general linear model was specified with regressors for “loss”, “stop” and “continue” events in the ON and OFF medication session. We added regressors modelling the scaled BOLD response to accumulated sum for each “continue” and “stop” event and the total sum lost for each “loss” event (i.e. parametric modulations). We included movement regressors (24 basis functions derived from spatial alignement (Friston et al., 1996)) as well as regressors modelling cardiac pulsation and respiration, using 3rd and 4th order aliased Fourier series respectively (Glover et al., 2000).

The first (single-subject) level general linear model (GLM) integrated the ON and OFF sessions in one model. We specified session-specific regressors for all relevant “continue”, “stop” and “loss” events as well as their parametric modulation with accumulated sum as separate regressors. We computed voxel-wise estimates for all regressors across both ON and OFF session (i.e. independent of medication) against zero as well as contrasting the ON and OFF session against each other. We then took these first level contrast maps to the second (group) level where we performed random-effects two-sample t-tests for A) both groups combined and B) ICD + and ICD - patients against each other and included CE as covariate.

**Fig. 2:**
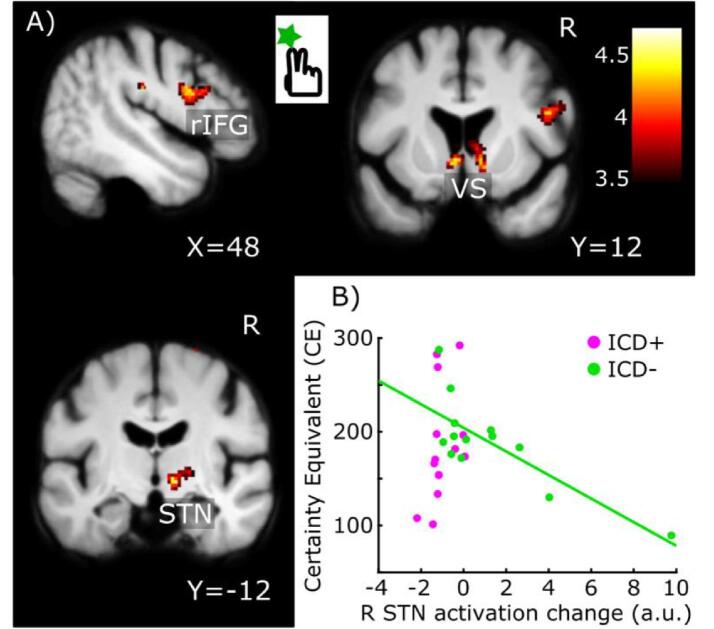
Between-group differences in “Continue” activity during sequential gambling independent of dopaminergic medication. The colour-coded statistical parametric t-score maps show reduced “continue” activity in PD patients with ICD irrespectively of accumulated sum or medication state (SPM thresholded at 0.001). Compared to patients without ICD, patients with ICD show less “continue” activity in the pars opercularis of right inferior frontal gyrgus (rIFG), right STN and ventral striatum/ventral caudate bilaterally. The scatter plot shows that “continue” activity in right STN scaled linearly with individual risk-taking attitudes during sequential gambling in patients without ICD (green circles, green line) but not in patients with ICD (purple circles). The more risk-aversive the choice behaviour (i.e., the lower the CE), the higher was the “continue” activity in right STN in the ICD - group. Please note that the correlation line is only illustrated in order to convey the linear scaling, the test for significance is established from the interaction effect based on the contrast images. (For interpretation of the references to colour in this figure legend, the reader is referred to the web version of this article.)

**Fig. 3:**
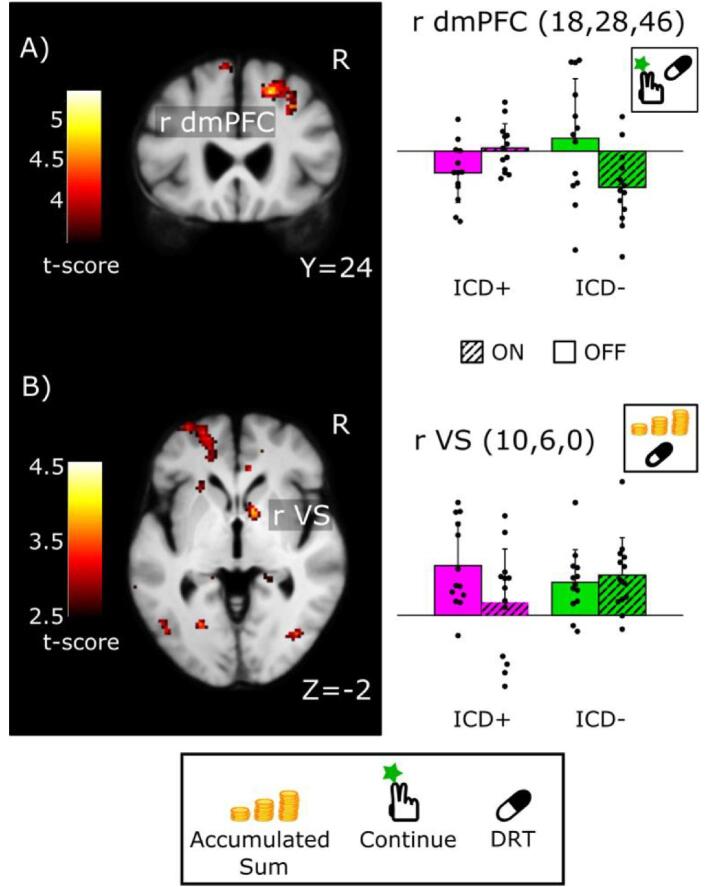
Brain regions where dopamine replacement therapy had group-specific effects on “Continue” activity during sequential gambling in PD patients with ICD relative to PD patients without ICD. In the right dorsomedial prefrontal cortex (dmPFC), ICD + patients showed a dopamine-related increase in “Continue” activity, whereas ICD - patients showed the opposite response pattern (SPM thresholded at 0.001 for illustrative purposes). B) In right ventral striatum (VS)/ventral caudate, dopamine replacement therapy attenuated the scaling of neural activity to accumulated sum during continued gambling in ICD + but not in ICD - patients (SPM thresholded at 0.005 for illustrative purposes). The left panels show the SPMs for the medication-by-group interaction contrast, while the right panels illustrate the estimated activation profiles (first Eigenvariate extracted for each subject from a 5 mm sphere around the peak-activation voxel, arbitrary units). Error bars correspond to SD. Note that the bar plots are only illustrated in order to convey the interaction effect, the test for significance is established from the interaction effect based on the contrast images.

We thus first aimed to replicate the increased activity of regions in the inhibitory control network during “continue” decisions with increasing accumulated sum across medication and groups as reported previously (Fig. 1D) (Meder et al., 2016). Our main hypotheses concerned activation differences during “continue” events where we first tested for group differences independent of medication (Fig. 2) and subsequently for group × medication interaction effects (Fig. 3).

Applying a Psycho-Physiological Interaction (PPI) analysis (Friston et al., 1997), we examined whether DRT would have a differential effect on functional connectivity of two cortical regions that are critical to inhibitory control, namely the pre-SMA and right IFG (Aron et al., 2007). The BOLD time-series from pre-SMA and rIFG (i.e., the physiological variable) were extracted from 5 mm spheres centred on activation peaks according to all participants pooled and multiplied after deconvolution with onsets of “continue” trials (i.e., the psychological variable). The resulting product was then convolved with the hemodynamic response function to test for a PPI.

To assess differential effects of DRT in ICD + and ICD -, we entered first-level contrast images corresponding to “ON > OFF” into a second-level random-effects analysis and computed a two-sample *t*-test to contrast ICD + and ICD -. The session-mean centred “Certainty Equivalent” (CE) was included as covariate of interest. Based on the Gaussian random field theory, topological correction for multiple comparisons was performed at the cluster-level using the family-wise error correction method as implemented in SPM. A corrected p-value of p < 0.05 was considered significant. We applied a voxel-wise threshold of p < 0.001 (uncorrected) to define cluster extent (Woo et al., 2014). This cluster forming threshold was sufficiently high to protect against inflated false-positive rates (Eklund et al., 2016).

Given our research hypotheses and based on the findings reported in previous studies (Frank et al., 2007, Rao et al., 2010, Voon et al., 2010, Voon et al., 2011), we applied small-volume correction (SVC) for VS, putamen and STN. For VS, we constructed the ROI by inclusively masking the common second-level effect of accumulated sum in “continue-to-gamble” trials using an uncorrected threshold of p < 0.001 with a bilateral anatomical mask including the nucleus accumbens and caudate head derived from the automated anatomical labelling (AAL) in the WFU PickAtlas toolbox (Tzourio-Mazoyer et al., 2002, Maldjian et al., 2003). For putamen, we used the common second-level main effect of “continue” trials thresholded at p < 0.001 (uncorrected) and applied an inclusive mask which included the bilateral putamen (AAL's putamen.nii). For STN, we used a probabilistic STN template derived from high-resolution 7 T structural scans (Forstmann et al., 2012) which was combined into a bilateral mask. For all ROI analyses, statistical threshold was set at p < 0.05 after small-volume correction, using voxel-level FWE correction (FWE_SVC_).

## 3 Results

### 3.1 Clinical and behavioural data

Groups did not differ significantly in terms of age, gender, handedness, disease duration, levodopa-equivalent daily dose or disease severity (Table 1). Mean QUIP scores differed significantly between groups (t_25_ = 4.83, p < 0.001, two-sample *t*-test).

In all participants, mean reaction times for continue-to-gamble decisions showed a positive linear relation with accumulated sum (*t*-test of regression coefficients against zero, t_25_ = 5.543, p < 0.001). A mixed-design ANOVA with within-subject factor medication and between-subject factor group showed no influence of medication or group on the linear increase in reaction time with accumulated sum (all p-values >0.25). Group or medication had also no effect on mean reaction times in “continue-to-gamble” trials and “stop” trials and no effect on mean CE (all p-values > 0.18).

### 3.2 Task-related brain activity across groups

Across both groups and both sessions, sequential gambling activated VS, the ventral head of caudate nucleus, anterior insula, bilateral lateral OFC and upper midbrain as well as bilateral inferior parietal lobules and secondary visual cortices. The cortico-subcortical response inhibition network showed a linear increase of “continue-to-gamble” activity with accumulated sum, including the pre-SMA, right IFG, and STN region (Fig. 1D; Supplementary Table 1).

### 3.3 Reduced brain activity during gambling in ICD + patients

ICD + patients showed a reduction of “continue-to-gamble” activity compared to patients without ICD (Fig. 2). Independent of the state of medication, the pars opercularis of rIFG (Z = 3.88, p = 0.032, x,y,z = 54,8,25), right VS/ventral caudate (Z = 3.52, p = 0.009 (FWE_SVC_), x,y,z = 10,12,-2), left VS/ventral caudate (Z = 3.08, p = 0.032 (FWE_SVC_), x,y,z = -6,14,2,), and right STN (Z = 3.79; p = 0.006 (FWE_SVC_), x,y,z = 12,-12,-4) showed a weaker activation during “continue-to-gamble” trials in the ICD + group relative to the ICD - group (Fig. 2a).

“Continue-to-gamble” activity in right STN showed an inverse linear relation with individual CE values in ICD - patients (Fig. 2b). The lower the “continue-to-gamble” activity in right STN, the longer patients continued to accumulate reward during sequential gambling as indexed by CE. Conversely, the higher the “continue-to-gamble” activity in right STN, the faster patients decided to refrain from accumulating reward. This relationship between “continue-to-gamble” activity in right STN and individual risk-taking during sequential gambling was not found in the ICD + group, resulting in a significant group-by-CE interaction (Z = 3.98, p = 0.003 (FWE_SVC_), x,y,z = 8,-16,-6) which was independent of medication.

### 3.4 Group-specific effects of medication state on accumulative gambling activity

In right dmPFC, DRT had a differential effect on “continue-to-gamble” activity (Fig. 3a). In the ICD + group, the right dmPFC displayed a medication-induced increase in “continue-to-gamble” activity, while the ICD - group showed the reverse response pattern (Z = 4.24, p = 0.006, x,y,z = 18,28,46). In right VS/ventral caudate, DRT diminished to the increase in “continue-to-gamble” activity with accumulated sum in ICD + group only (Fig. 3b; Z = 3.20, p = 0.028 (FWE_SVC_), x, y, z = 10, 6, 0)

### 3.5 Functional connectivity changes

**Fig. 4:**
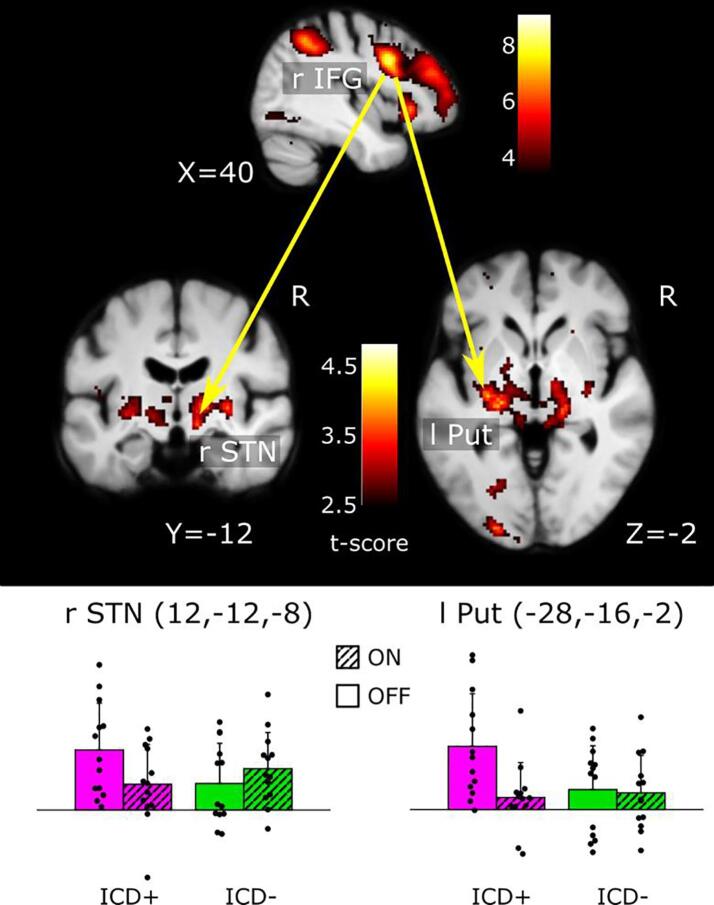
Functional connectivity analysis. Psycho-Physiological-Interaction (PPI) analysis reveals prefrontal-subcortical reduction in functional connectivity during the on-medication state in PD patients with ICD. Dopamine replacement therapy was associated with a reduced influence of right inferior frontal gyrus (rIFG) on neural activity in left posterior putamen (Put) and right subthalamic nucleus (STN) during accumulative gambling. The SPMs (top panel) show the medication-by-group effect on functional connectivity. PPI analysis was performed at the subject-level using the rIFG as source region and treating “continue-to-gamble” events as experimental context. The column graphs illustrate the estimated context-dependent functional connectivity in the ON− and OFF-medication state for the ICD + and ICD - group. The first eigenvariate was extracted for each subject from a 5 mm sphere around the peak-activation voxel, arbitrary units. Error bars correspond to SD. Note that the bar plots are only illustrated in order to convey the interaction effect, the test for significance is established from the interaction effect based on the contrast images. SPMs are thresholded at p < 0.005 (uncorrected) for illustrative purposes.

Using the rIFG as seed region, PPI analysis revealed that DRT resulted in a group-specific reduction of functional connectivity between rIFG and left posterior putamen (Z = 3.43, p = 0.039 (FWE_SVC_), x, y, z = −28, −16, −2) and right STN (Z = 3.18, p = 0.042 (FWE_SVC_), x, y, z = 12, −12, −8). This effect was driven by a DRT-related decrease in functional connectivity of the right IFG during “continue-to-gamble” decisions with left posterior putamen and right STN in the ICD + group (Fig. 4).

A PPI analysis seeding in the pre-SMA did not yield any group or interaction effects, but there was a significant main effect of medication when all PD patients were considered together. During the OFF-medication state, pre-SMA showed stronger bilateral functional connectivity with the STN (right STN: p = 0.039 (FWE_SVC_), x, y, z = 12, −12, 0, z = 3.25; left STN: p = 0.042 (FWE_SVC_), x, y, z = −8, −12, −12, Z = 3.23) and VS (right VS: p = 0.005 (FWE_SVC_), x, y, z = −12, 14, −2, Z = 3.72; left VS: p = 0.008 (FWE_SVC_), x, y, z = 14, 16, 0, Z = 3.60). The OFF-medication state was also associated with a stronger cortico-cortical functional connectivity between pre-SMA and clusters in medial prefrontal cortex and adjacent anterior cingulate cortex (Z = 4.47, p < 0.001, x, y, z = 14, 42, 18), right inferior temporal gyrus (Z = 4.32, p = 0.048, x, y, z = 60, −38, −12), and left precuneus (Z = 3.96, p = 0.007, x, y, z = −10, −66, 34) as well as stronger cortico-cerebellar functional connectivity between pre-SMA and the right and left superior cerebellar lobule (right: Z = 4.03, p = 0.001, x,y,z = 8,–82,-26; left: Z = 4.02, p = 0.001, x, y, z = −32, −50, −24; see Supplementary information S3).

## 4 Discussion

**Fig. 5:**
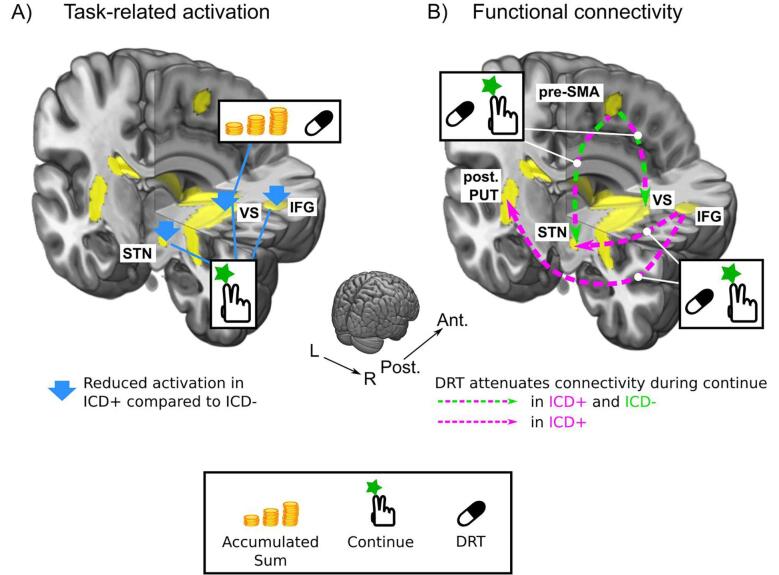
Synopsis of the alterations in task-related activity (A) and connectivity (B) in PD patients with ICD (ICD +) relative to PD patents without ICD (ICD -). A) Differences in task-related activity between the ICD + and ICD - groups. During Continue trials, activity in right inferior frontal gyrus (rIFG), ventral striatum (VS) and subthalamic nucleus (STN) was decreased in the ICD + group compared to the ICD - group. During continue-to-gamble trials, the linear scaling of task-related activity in the VS with the accumulated sum was diminished by Dopamine Replacement Therapy (DRT) in the ICD + group only. B) Differences in task-related connectivity between ICD + and ICD - groups. In both groups, DRT diminished functional connectivity during continue trials between pre-supplementary motor area (pre-SMA) and VS and pre-SMA and STN. In ICD + but not ICD - patients, functional connectivity was also diminished between the rIFG and the posterior putamen and STN during continue-to-gamble trials.

We found several links between impaired impulse control and altered circuit activity in prefronto-basal ganglia circuits in Parkinson’s disease (Fig. 5). When engaging in sequential gambling, patients with disordered impulse control showed functional changes in fronto-subthalamic inhibitory network as well as striatal motivational-limbic areas relative to patients without problems with impulse control. Regardless of the state of medication, the presence of ICD was associated with reduced “continue-to-gamble” activity in core regions of the fronto-subthalamic inhibitory network. ICD + patients also showed a reduction in functional connectivity between STN activity and individual risk-taking behaviour independent of the state of medication. Dopamine substitution attenuated functional cortico-subcortical functional connectivity in the fronto-subthalamic inhibitory network in both patient groups, reducing functional connectivity of pre-SMA with STN and striatum. Only in ICD + patients, dopamine replacement also suppressed functional connectivity of right IFG with STN and striatum. We first discuss ICD-related alterations in the activation of inhibitory networks during sequential gambling and thereafter alterations in striatal activation.

### 4.1 ICD-related changes in action control networks

The gambling task required patients to continuously trade-off between prospect of more reward and higher losses. Confirming our previous results in healthy individuals (Meder et al., 2016), patients with Parkinson’s disease showed a linear increase of event-related “continue-to-gamble” activity with the accumulated gain during the gambling rounds in pre-SMA, right IFG, striatum and STN (see Supplementary information 1 and Fig. 1D), along with a gradual increase in mean response times. This network has previously been implicated in reactive and proactive stopping, response switching and motivational tuning of action inhibition (Aron et al., 2014, Herz et al., 2014, Aron et al., 2007, Jahfari et al., 2009, Neubert et al., 20107, Rae et al., 2015). Regardless of DRT, event-related activity in the rIFG pars opercularis, right STN, and bilateral VS/ventral caudate was weaker during “continue”-trials in ICD + patients compared to ICD - patients (Fig. 2, Fig. 5). Beyond some studies implicating differences in the activity of response-control regions such as the ACC in ICD (van Eimeren et al., 2010, Hammes et al., 2019), this finding indicates a reduced engagement of both the inhibitory control network (i.e., right STN and right IFG) and reward processing network (i.e., right and left VS), in sequential gambling in PD patients with ICD, showing that both processes are altered in PD patients with ICD (Mosley et al., 2019, Paz-Alonso et al., 2020).

During sequential gambling, we found a between-group difference in the linear relationship between “continue-to-gamble” activity in right STN and risk-taking behaviour as reflected by the CE value. In the ICD - group, STN activity scaled negatively with individual CE values, indicating that a stronger engagement of the STN was associated with less risk taking. This relationship was not present in the ICD + group (Fig. 2). Similarly, a recent structural MRI study observed a linear relationship between structural connectivity of a “reward evaluation” circuit and risky gambling in the ICD - group, but not in the ICD + group, in the absence of differences in gambling behaviour between the groups (Fumagalli et al., 2015, Mosley et al., 2019, Rosa et al., 2013). Previous electrophysiological studies found risky strategies in gambling to manifest differently in STN in ICD + patients (Fumagalli et al., 2015, Rosa et al., 2013). Our finding suggests that STN activity relates to how soon ICD - patients stop their risky engagement, but that this relationship is disturbed in ICD + patients.

The state of dopaminergic medication influenced cortico-subcortical functional connectivity within the inhibitory control network. Using the pre-SMA as seed region, PPI analyses revealed that dopamine replacement reduced functional connectivity between the pre-SMA and the STN and ventral striatum/ventral caudate bilaterally in both patient groups. For the rIFG, our PPI analysis yielded a weakening of functional connectivity with the right STN and the putamen in the ON medication state, but only in the ICD + group (Fig. 4, Fig. 5). Together, our findings point to a “dual-hit mechanism” in patients with ICD. While the normal response to dopamine in PD entails a reduced functional connectivity of the pre-SMA with the STN and VS, patients with ICD show an additional weakening of the influence of right IFG on STN and putamen. In the absence of a healthy control group, we cannot conclude whether the reduced connectivity between pre-SMA and STN/VS upon dopamine medication is specific to PD or whether it would also occur in healthy participants.

Our connectivity results extend two resting-state connectivity studies, reporting a diminished fronto-striatal connectivity at rest in PD patients with ICD (Carriere et al., 2015, Cilia et al., 2011). Structurally, this may be underpinned by diminished structural connectivity within the inhibitory network (Mosley et al., 2019). Another recent study found that in the context of negative feedback in the Iowa Gambling Task, functional connectivity between right VS and rIFG increased with increasing impulsivity (QUIP-measure) in ICD-patients ON DRT (Paz-Alonso et al., 2020). Taken together, these data point to a critical role of connectivity within and between inhibitory and reward networks in ICD, however the directionality of changes in connectivity possibly depends on gambling context (anticipation or feedback phase). The right-lateralization of the observed changes are in line with the general notion of a predominantly right-lateralized inhibitory action-control network (Aron et al., 2007, Rae et al., 2015). Interestingly, a recent study in patients with PD demonstrated detrimental effects of right STN deep-brain stimulation (DBS) on inhibitory control, further emphasizing the importance of this nucleus (Mosley et al., 2018).

The present study is the first to explore how DRT modulates connectivity in ICD. Our PPI findings suggest that DRT acts as a pharmacological “switch”, attenuating information transfer within the two main cortico-subcortical pathways in ICD + patients. This switch may be permissive for the emergence of impulsive actions and thus may serve as an important neurobiological trigger of ICD (Carriere et al., 2015, Cilia et al., 2011).

Importantly, the treatment-induced effects on reward-related activity in ICD + reported here occurred in the absence of a behavioural group difference in risk-taking attitude (CE) or response-slowing by stake. The absence of a difference in risk-taking in our study may be attributed to a relatively low number of ICD + patients, a substantial inter-individual variation in task-related risk-taking attitude, and the fact that their ICD-behaviours were not confined to gambling or shopping.

Perhaps a behavioural difference would have emerged, had the gambling task offered a faster-paced environment with the possibility to individually change bet-sizes, as in real gambling machines. In our paradigm, the increase in bet sizes was only gradual and the time between each gamble was rather long (3.5–5.5 s). However, similar to our results, a recent structural conenctivity study used a self-paced gambling task with multiple options to increase bet size and found interactions between structural connectivity in both, the reward and the inhibitory network, and impulsive behaviour, dependent on ICD status, but no main effect of ICD on risk-taking behaviour in the task (Mosley et al., 2019). Neither was risk-taking behaviour different between ICD + and ICD - patients in two fMRI studies using gambling tasks (Rao et al., 2010, Paz-Alonso et al., 2020). This facilitates the interpretation of our fMRI results because differences between group cannot be attributed to differences in task-related behaviour. Therefore, the observed differences in task-related regional activity and inter-regional connectivity reveal a phenotypical neural profile of ICD across different ICD-subtypes that is already expressed when ICD patients engage in “subsyndromal” behaviour.

Despite of the lack of behavioural differences between ICD + and ICD - patients in our sequential gambling task, we argue that the cortico-STN hyper-direct pathway is important for self-regulatory (i.e. not externally cued) inhibitory control. This has been stressed by several functional neuroimaging studies on healthy volunteers, implicating that intact cortico-subthalamic connectivity is critical for solving choice conflicts or task difficulty (Frank et al., 2015, Zavala et al., 2014) as well as response inhibition in contexts of reward and risk (Herz et al., 2014, Meder et al., 2016). Further evidence comes from a recent DBS study in patients with PD (Irmen et al., 2019). In that study, bilateral DBS of STN normalized the overly risk-averse behaviour of PD patients. The more the DBS preferentially targeted the motor territory, the more the patients showed a normal trade-off between risk and reward during sequential gambling (Irmen et al., 2019).

### 4.2 ICD-related changes in reward processing

DRT also attenuated the reward and risk-related responses during gambling differently in the two groups (Fig. 3). In patients with ICD, DRT reduced the VS response to accumulated sum relative to PD patients without ICD. In our task, accumulated sum represents an increase in risk as well as an increase in expected reward, both of which have been shown to engage the VS (Preuschoff et al., 2006, Schultz, 2010). Thus, the observed VS response may reflect neural activity processing of gradual increases in expected reward, risk magnitude, or both. Previous literature suggests that the reduced activation of VS seen in the ICD group is most likely related to an attenuation of risk-processing. Supporting the latter possibility, a pharmacological fMRI study with a single-shot gambling task with varying risk levels found a reduction of VS activity in PD patients with ICD due to dopaminergic medication (Voon et al., 2011). This interpretation is also in agreement with another neuroimaging study examining brain activity during the Balloon Analogue Risk Task in the ON medication state (here, however, the deactivation was also observed during rest) (Rao et al., 2010). The other possibility, namely that altered VS activity is related to processing of expected reward with the gradual increase in accumulated sum, is less likely, because a previous neuroimaging study would predict an opposite relationship (Voon et al., 2010). In that study, DRT increased VS activity related to anticipated rewards in ICD + patients (Voon et al., 2010). Since the scaling of VS activity in proportion with accumulated sum was attenuated rather than increased in our study, we hypothesize that the reduced increase in VS activity with accumulated sum reflects a reduction in processing risk and potential loss rather than changes in computing expected reward. Thus, the present finding adds evidence to the notion that in PD patients with ICD (Voon et al., 2011), DRT may compromise neural encoding of risk in the VS, facilitating risk-prone behaviours. This hypothesis is also supported by a recent multimodal neuroimaging study which showed a negative correlation between dopamine synthesis capacity in the VS and severity of impulsive-compulsive behavior (Hammes et al., 2019).

We also found as mentioned above a diminished activity in VS in ICD + during “continue-to-gamble” decisions as such, irrespective of accumulated gains and this diminution was present in ON as well as OFF, i.e. as a “trait-phenomenon” of ICD +. Thus, our results suggest that DRT compromises the increase in VS activity under increasing risk on top of a generally reduced response during risky actions per se.

Thus, the pattern of changes points to an underlying disposition for ICD in the reward network (including VS) and the inhibitory control network (rSTN, rIFG, preSMA), in accordance with other recent studies (Imperiale et al., 2018, Mosley et al., 2019). Together, these findings may serve as first steps towards a neuroimaging-based identification of PD patients susceptible to developing ICD.

In conclusion, we describe a combination of abnormalities in the functional brain networks underpinning action inhibition and reward processing in a group of PD patients with ICD and how these are modulated by DRT. Our results point to neurobiological underpinnings of ICD that involve cortico-subthalamic action inhibition and striatal reward-risk processing. It should be noted that we observed significant differences in head movement between the two groups. However, the mean difference was only about 0.1 mm and we included motion regressors in the design matrix. Thus, given the nature of the connectivity and interaction results, we deem the difference unlikely to influence the current findings. The observed neural differences emerged in a mixed cohort of ICD + patients and in the absence of behavioural differences in task performance between the ICD + and ICD - group. The joint functional alteration of inhibitory and striatal networks may thus constitute a common neural “*endo*-phenotype” of ICD.

## CRediT authorship contribution statement

**Brian N. Haagensen:** Conceptualization, Methodology, Investigation, Formal analysis, Writing - original draft, Writing - review & editing. **Damian M. Herz:** Conceptualization, Methodology, Investigation, Formal analysis, Writing - review & editing. **David Meder:** Conceptualization, Software, Methodology, Formal analysis, Writing - review & editing. **Kristoffer H. Madsen:** Investigation, Formal analysis, Writing - review & editing. **Annemette Løkkegaard:** Investigation, Formal analysis, Writing - review & editing. **Hartwig R. Siebner:** Conceptualization, Methodology, Writing - review & editing, Resources, Writing - review & editing, Supervision, Project administration, Funding acquisition.

## Declaration of Competing Interest

The authors declare the following financial interests/personal relationships which may be considered as potential competing interests: Hartwig R. Siebner has received honoraria as speaker from Sanofi Genzyme, Denmark and Novartis, Denmark, as consultant from Sanofi Genzyme, Denmark and as senior editor (NeuroImage) from Elsevier Publishers, Amsterdam, The Netherlands. He has received royalties as book editor from Springer Publishers, Stuttgart, Germany. Annemette Løkkegaard has received financial suppport from Medtronic and AbbVie A/S to participate in scientific meetings as well as a honorarium as speaker from AbbVie A/S. All other authors report no conflict of interest.
