# Supplementary material for: Linking brain activity during sequential gambling to impulse control in Parkinson's disease
Source: Neuroimage Clin. 2020 Jun 25;27:102330. doi: 10.1016/j.nicl.2020.102330 (PMC7369593; doi:10.1016/j.nicl.2020.102330)
Supplement: Supplementary data 1 [file mmc1.docx]

**SUPPLEMENTARY INFORMATION S1**

| Region | p-value | Peak Z-score | Peak MNI coordinate | | |
| --- | --- | --- | --- | --- | --- |
|  |  |  | x | y | z |
| **i) Increases in regional neural activity during "Continue-to-gamble" trials** |  |  |  |  |  |
| **Cluster 1 (2713)** | < 0.001 |  |  |  |  |
| L. insula |  | 5.84 | -34 | 2 | 16 |
| L. operculum |  | 5.61 | -46 | -20 | 18 |
| L. posterior putamen |  | 5.53 | -28 | -8 | 14 |
| **Cluster 2 (1084)** | < 0.001 |  |  |  |  |
| R. ant. putamen |  | 5.69 | 26 | 4 | 4 |
| R. post. putamen |  | 4.57 | 28 | -16 | 6 |
| R. insula |  | 4.34 | 40 | 2 | 14 |
| **Cluster 3 (3862)** | < 0.001 |  |  |  |  |
| R. occipital lobe/cerebellum |  | 5.03 | 28 | -72 | -12 |
| **Cluster 4 (949)** | < 0.001 |  |  |  |  |
| L. cerebellum/occipital lobe |  | 4.82 | -24 | -62 | -22 |
| **ii) Increases in “Continue-to-gamble” activity in proportion with accumulated sum** |  |  |  |  |  |
| **Cluster 1 (1085)** | < 0.001 |  |  |  |  |
| R. ant. insula |  | 5.78 | 32 | 26 | -2 |
| R. ventral striatum |  | 5.15 | 8 | 12 | 4 |
| **Cluster 2 (5229)** | < 0.001 |  |  |  |  |
| R. inf. front. gyrus |  | 5.56 | 40 | 10 | 32 |
| R. dorsolat. PFC |  | 5.01 | 44 | 42 | 26 |
| R. pre-SMA |  | 4.95 | 4 | 18 | 52 |
| R. sup. front. gyrus |  | 4.21 | 34 | 8 | 64 |
| R. mid. front. gyrus |  | 3.97 | 34 | -2 | 50 |
| R. OFC |  | 3.88 | 26 | 50 | -12 |
| **Cluster 3 (1027)** | < 0.001 |  |  |  |  |
| L. ant. insula |  | 5.49 | -32 | 26 | 2 |
| L. ventral striatum |  | 4.29 | -10 | 6 | 2 |
| **Cluster 4 (1043)** | < 0.001 |  |  |  |  |
| L. occipital lobe |  | 5.42 | -26 | -98 | 0 |
| **Cluster 5 (2505)** | < 0.001 |  |  |  |  |
| L. inf. front. gyrus |  | 5.02 | -38 | 10 | 30 |
| L. dorsolat. PFC |  | 4.46 | -46 | 26 | 30 |
| L. sup. front. gyrus |  | 3.74 | -26 | -8 | 54 |
| L. OFC |  | 3.39 | -36 | 58 | -10 |
| **Cluster 6 (1482)** | < 0.001 |  |  |  |  |
| R. inf. parietal lobe |  | 4.96 | 40 | -52 | 46 |
| Cluster 7 (1734) | < 0.001 |  |  |  |  |
| L. inf. parietal lobe |  | 4.6 | -34 | -46 | 38 |
| **Cluster 8 (481)** | 0.003 |  |  |  |  |
| R./L. midbrain (Subst.nigra/STN) |  | 4.27 | 6 | -20 | -8 |
| **Cluster 9 (371)** | 0.010 |  |  |  |  |
| R. occipital lobe |  | 4.11 | 26 | -96 | 6 |

**SUPPLEMENTARY INFORMATION S2**

| Region | p-value | Peak Z-score | Peak MNI coordinate | | |
| --- | --- | --- | --- | --- | --- |
|  |  |  | x | y | z |
| **i) Increases in regional neural activity during "Stop" trials** |  |  |  |  |  |
| **Cluster 1 (82944)** | < 0.001 |  |  |  |  |
| R. inf. parietal lobe |  | 7.73 | 42 | -46 | 42 |
| L. ant. insula/inf. front. gyrus |  | 7.52 | -30 | 20 | 4 |
| L. sup. cerebellar lobule |  | 7.12 | -24 | -62 | -24 |
| L./R. ant. cingulate cortex |  | 7.06 | -8 | 24 | 38 |
| L. thalamus |  | 7.05 | -10 | -14 | 6 |
| L. putamen/operculum |  | 7.01 | -32 | -2 | 12 |
| R. ant. insula/inf. front. gyrus |  | 6.97 | 32 | 24 | -4 |
| R./L. pre-SMA |  | 6.90 | 8 | 20 | 52 |
| L. caudate |  | 6.82 | -16 | 10 | -2 |
| **Cluster 2 (316)** | 0.018 |  |  |  |  |
| R./L. Pons |  | 4.47 | 4 | -18 | -34 |
| **ii) Increases in regional neural activity during "Loss" trials** |  |  |  |  |  |
| **Cluster 1 (2479)** | < 0.001 |  |  |  |  |
| L.ant. insula/putamen |  | 6.53 | -36 | 20 | -4 |
| **Cluster 2 (2654)** | < 0.001 |  |  |  |  |
| R. ant. insula/putamen |  | 6 | 28 | 10 | 0 |
| **Cluster 3 (1970)** | < 0.001 |  |  |  |  |
| R. inf. parietal lobe |  | 5.84 | 46 | -50 | 38 |
| **Cluster 4 (1536)** | < 0.001 |  |  |  |  |
| R. inf. temporal gyrus |  | 5.71 | 64 | -34 | -8 |
| **Cluster 5 (4098)** | < 0.001 |  |  |  |  |
| R./L. dorsal ant. cingulate cortex |  | 5.47 | 6 | 28 | 42 |
| R. dorsolateral PFC |  | 4.84 | 10 | 42 | 34 |
| R. pre-SMA |  | 4.32 | 10 | 22 | 58 |
| **Cluster 6 (7310)** | < 0.001 |  |  |  |  |
| R. cerebellum/occipital lobe |  | 5.24 | 46 | -68 | -28 |
| **Cluster 7 (868)** | < 0.001 |  |  |  |  |
| R./L. precuneus |  | 4.81 | 10 | -64 | 50 |
| **Cluster 8 (409)** | 0.005 |  |  |  |  |
| L. hippocampus |  | 4.47 | -20 | -4 | -32 |
| **Cluster 9 (451)** | 0.003 |  |  |  |  |
| L. inf. parietal lobe |  | 4.33 | -36 | -60 | 42 |
| **ii) Increases in “Loss” activity in proportion with loss-size** |  |  |  |  |  |
| **Cluster 1 (1542)** | < 0.001 |  |  |  |  |
| L. ant. insula/inf. front. gyrus |  | 5.58 | -30 | 22 | 6 |
| **Cluster 2 (2618)** | < 0.001 |  |  |  |  |
| R. pre-SMA |  | 5.35 | 12 | 14 | 50 |
| R. dorsal ant. cingulate cortex |  | 4.74 | 12 | 30 | 28 |
| **Cluster 3 (2872)** | < 0.001 |  |  |  |  |
| R. caudate nucleus |  | 5.26 | 10 | 2 | 12 |
| R./L. post. cingulate cortex |  | 4.74 | 2 | -30 | 30 |
| R. thalamus |  | 4.64 | 8 | -10 | 14 |
| R. post. caudate |  | 4.46 | -16 | -4 | 14 |
| L. caudate nucleus |  | 4.28 | -12 | 2 | 4 |
| L. putamen |  | 3.99 | -22 | 0 | 8 |
| **Cluster 4 (1373)** | < 0.001 |  |  |  |  |
| R. ant. insula/inf. frontal gyrus |  | 5.21 | 40 | 28 | -8 |
| **Cluster 5 (684)** | < 0.001 |  |  |  |  |
| R. precuneus |  | 5.18 | 8 | -68 | 54 |
| R. sup. parietal lobe |  | 3.6 | 28 | -72 | 44 |
| **Cluster 6 (1399)** | < 0.001 |  |  |  |  |
| R. occipital lobe |  | 4.65 | 30 | -58 | -14 |
| **Cluster 7 (368)** | 0.004 |  |  |  |  |
| L. occipital lobe/cerebellum |  | 4.42 | -40 | -64 | -18 |
| **Cluster 8 (263)** | 0.019 |  |  |  |  |
| R. mid. front. gyrus |  | 4.27 | 20 | 50 | 22 |

There were no significant differences in activity between the ICD+ and the ICD- group for stop or loss events.

**SUPPLEMENTARY INFORMATION S3**


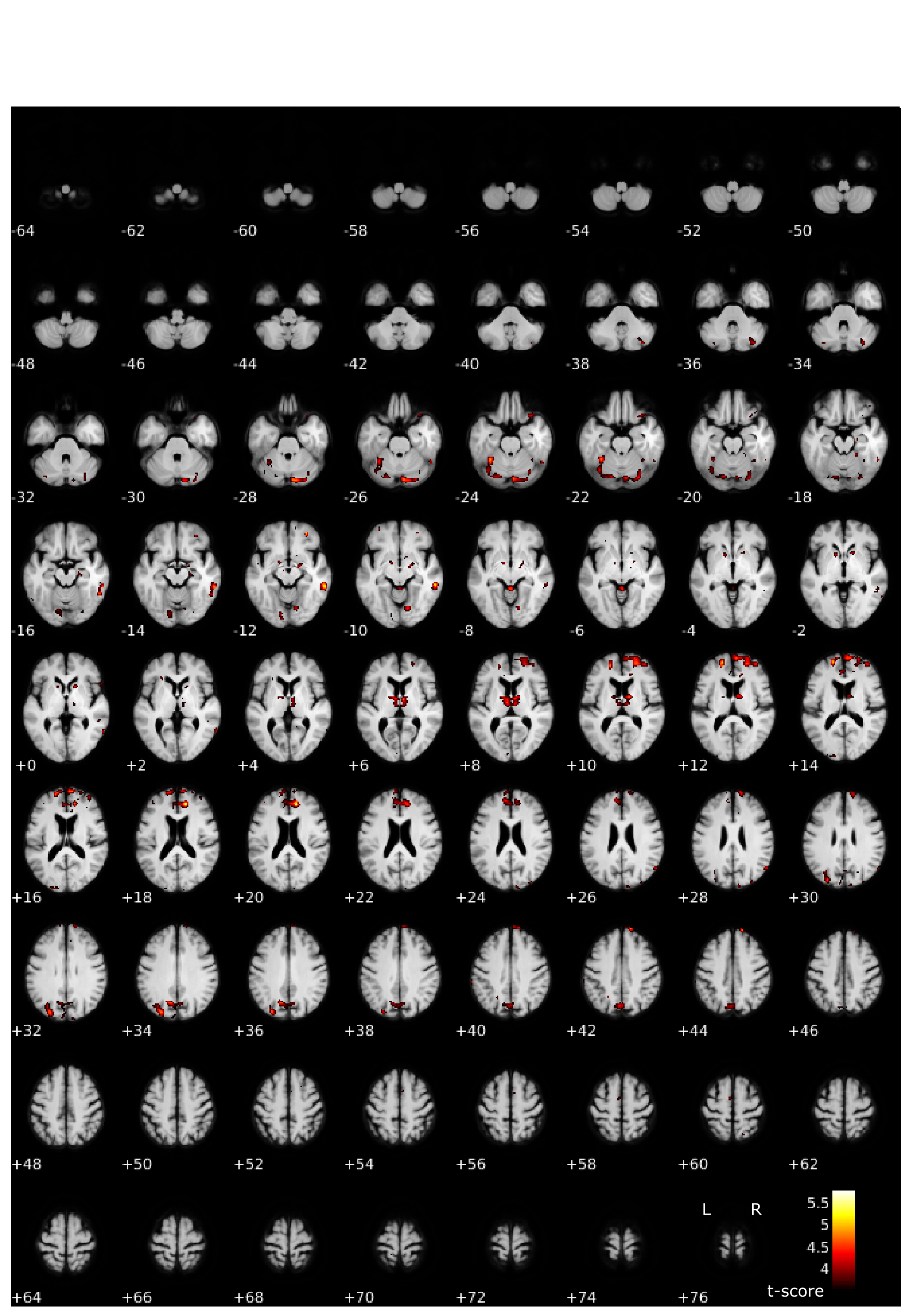


**Supplementary Figure showing the results of a PPI with pre-SMA as seed region revealing a significant main effect of medication when all PD patients were considered together.** During the OFF-medication state, pre-SMA showed stronger bilateral functional connectivity with the STN (right STN: p=0.039 (FWE SVC), x,y,z=12,-12,0, z=3.25; left STN: p=0.042 (FWE SVC), x,y,z=-8,-12,-12, Z = 3.23) and VS (right VS: p=0.005 (FWE SVC), x,y,z =-12,14,-2, Z=3.72; left VS: p=0.008 (FWE SVC), x,y,z=14,16,0, Z=3.60), medial prefrontal cortex and adjacent anterior cingulate cortex (Z=4.47, p<0.001, x,y,z=14,42,18), right inferior temporal gyrus (Z=4.32, p=0.048, x,y,z=60,-38,-12), and left precuneus (Z=3.96, p=0.007, x,y,z=-10,-66,34) as well as right and left superior cerebellar lobule (right: Z=4.03, p=0.001, x,y,z=8,‑82,-26; left: Z=4.02, p = 0.001, x,y,z=-32,-50,-24).
